# Environmental Surveillance of Poliovirus in the Democratic Republic of the Congo: Sensitivity and Site Productivity, 2017–2025

**DOI:** 10.3390/pathogens15090917

**Published:** 2026-08-31

**Authors:** Vanessa Bakumba-Kiulu, Paul Tshiminyi-Munkamba, Trésor Kabeya-Mampuela, Meris Matondo-Kuamfumu, Grace Kashitu-Mujinga, Junior Bulabula-Penge, Elisabeth Pukuta-Nsimbu, Elysabeth Muyamuna-Mulongo, Yvonne Lay Mowele, Jean Claude Mukangala-Changa-Changa, Eddy Kinganda-Lusamaki, Narcisse Yogolelo-Riziki, Antoine Nkuba-Ndaye, Steve Ahuka-Mundeke

**Affiliations:** 1 Service of Microbiology, Department of Medical Biology, Kinshasa Teaching School of Medicine, Faculty of Medicine, University of Kinshasa, Kinshasa BP 125, Democratic Republic of the Congo; tresorkabens16@gmail.com (T.K.-M.);; 2 Department of Virology, Institut National de Recherche Biomédicale (INRB), Kinshasa 01204, Democratic Republic of the Congo; doctapaul2010@gmail.com (P.T.-M.);; 3 Faculty of Medicine, Université Protestante au Congo, Kinshasa 01212, Democratic Republic of the Congo; 4 Department of Ecoepidemiology, Institute of Tropical Medicine (Nekken), Nagasaki University, Nagasaki 852-8523, Japan; 5 TransVIHMI, Institut de Recherche pour le Développement, Université de Montpellier, 34394 Montpellier, France

**Keywords:** poliovirus, environmental surveillance, Democratic Republic of the Congo

## Abstract

Background: Environmental surveillance (ES) of wastewater was introduced by the Global Polio Eradication Initiative (GPEI) to improve the sensitivity of acute flaccid paralysis (AFP) surveillance. In the Democratic Republic of the Congo (DRC), ES for poliovirus has been implemented since 2017. This study assessed its sensitivity and productivity. Methods: A retrospective descriptive study using ES for poliovirus data collected in the DRC between September 2017 and June 2025. Wastewater samples were analyzed at the National Institute for Biomedical Research (INRB), using a standardized algorithm that included concentration, cell culture, real-time reverse transcription polymerase chain reaction (rRT-PCR), and VP1 sequencing. Site sensitivity was assessed by productivity, defined as a viral isolation rate ≥ 50%. Results: A total of 2491 wastewater samples were collected. ES expanded from 6 sites in 3 provinces to 27 sites across 8 provinces. Overall site productivity was 33.9% (846/2491 cultures positive for enteroviruses), showing significant variability between sites and over time, which sometimes led to site closures. Non-polio enteroviruses were the most commonly isolated viruses. Among poliovirus isolates, Sabin-like strains (types 1, 2, and 3), novel oral poliovirus vaccine type 2 (nOPV2), and vaccine-derived poliovirus type 2 (VDPV2) were identified, with VDPV2 representing 13.1%. No wild poliovirus was detected. Conclusion: ES for poliovirus has expanded in the DRC, helping detect vaccine-derived poliovirus circulation and confirming the absence of wild poliovirus. Despite ongoing operational and logistical challenges causing fluctuations in site productivity, this study emphasizes the vital role of ES as a supplement to AFP surveillance.

## 1. Introduction

Poliomyelitis is an acute infectious disease caused by polioviruses, which are non-enveloped, positive-sense, single-stranded ribonucleic acid (RNA) enteroviruses [1,2]. In approximately 0.1–1% of infections, the virus can cause irreversible motor impairment, manifested as acute flaccid paralysis (AFP) [3]. Polioviruses are primarily transmitted via the fecal–oral route and are excreted in the stool of infected individuals for several weeks, often without clinical symptoms. In settings with poor sanitation, the virus may contaminate the environment and can be detected in sewage and wastewater, reflecting the risk of silent transmission by asymptomatic carriers [4,5].

To eliminate this disabling disease, the Global Polio Eradication Initiative (GPEI) was launched in 1988 by the World Health Assembly (WHA). The main strategies included mass immunization using the oral poliovirus vaccine (OPV), surveillance of AFP cases, investigation of poliovirus in stool samples from AFP cases and their contacts under five years of age, and destruction of isolated viral strains as part of containment measures [3]. These interventions led to remarkable progress, including a reduction of more than 99% in global poliomyelitis cases. Two of the three wild poliovirus serotypes were declared eradicated, namely WPV2 in 2015 and WPV3 in 2019, leaving only WPV1 endemic in Pakistan and Afghanistan [3,6,7,8,9].

However, despite these substantial achievements toward eradication, the risk of WPV re-emergence and outbreaks associated with vaccine-derived polioviruses (VDPVs) remains a major public health concern, particularly in settings with low vaccination coverage. In 2019, the World Health Organization (WHO) reported 41 circulating VDPV (cVDPV) emergences associated with AFP cases across 18 countries, followed by 34 emergences in 25 countries in 2020 [7]. In 2022, imported WPV1 cases were confirmed in Malawi and Mozambique [9,10]. In 2024, 297 cVDPV2 cases were reported worldwide, with the highest numbers recorded in Nigeria (98 cases), Ethiopia (43 cases), Chad (39 cases), Yemen (37 cases), Niger (16 cases; 5.4%), and the Democratic Republic of the Congo (15 cases). By May 2025, a total of 44 WPV1-positive cases had been confirmed in Afghanistan and Pakistan, in addition to 49 cVDPV2 AFP cases and 57 positive environmental surveillance detections globally [11].

In response to the ongoing risk of poliovirus resurgence and recurrent VDPV outbreaks, environmental surveillance for poliovirus has been progressively implemented and expanded in several endemic countries with support from the GPEI, including the DRC. This virological environmental surveillance approach enhances the sensitivity of AFP surveillance and serves as an early warning indicator of potentially hundreds or thousands of silent poliovirus infections during outbreaks or in endemic settings. Environmental surveillance was introduced at an advanced stage of the eradication process because it also contributes to poliovirus containment and supports optimal monitoring of virus elimination. Its objective is to strengthen eradication efforts by monitoring poliovirus circulation and detecting the emergence of VDPVs in the environment [4,7].

Environmental surveillance (ES) involves analyzing wastewater collected from selected sites within defined health areas to detect poliovirus circulation. Site selection follows the recommendations outlined in the field guidelines for poliovirus environmental surveillance. These recommendations are based on three main principles: (i) identification of areas at high risk of viral circulation, (ii) balancing sensitivity with operational feasibility, and (iii) close collaboration between field teams, program focal points, and reference laboratories. One of the key site performance indicators is the detection of enteroviruses (polioviruses or non-polio enteroviruses) in at least 50% of collected samples, reflecting the surveillance system’s sensitivity in line with WHO standards. Site selection, therefore, takes into account both epidemiological relevance and the size of the population served. A sampling site located in a high-risk area is considered appropriate when the sewage network draining into the site covers a population ranging from 100,000 to 300,000 individuals [4,7].

In the DRC, poliomyelitis surveillance began in 1997 through AFP surveillance activities. Although the country was certified free of wild poliovirus by the WHO in November 2015, it remains vulnerable to recurrent cVDPV outbreaks, as previously described. Environmental surveillance was introduced in 2017 as part of the global expansion plan for poliovirus environmental surveillance.

The increasing number of VDPV detections across several provinces of the country raises concerns about the virus’s silent circulation in the environment. The implementation of environmental surveillance, therefore, represents a crucial step toward strengthening the national surveillance system. However, since the introduction of this strategy, no comprehensive evaluation of environmental surveillance performance in the DRC, particularly regarding site productivity, has been conducted. Previous studies assessing AFP and environmental surveillance performance indicators have shown substantial variations across countries and WHO regions [7,12].

Within this context, the present study aimed to provide an overview of poliovirus environmental surveillance in the DRC to assess its sensitivity and its contribution to poliovirus eradication.

## 2. Materials and Methods

### 2.1. Study Design and Setting

This retrospective descriptive study examined environmental virological surveillance of poliovirus in the Democratic Republic of the Congo (DRC). The study used routine surveillance data collected by the national poliovirus environmental surveillance (ES) reference laboratory from September 2017 through June 2025.

During this period, 29 sampling sites located across 8 provinces were used to collect wastewater samples. The types of sewage plantor system consisted of open canals for 27 sites and a closed canal for 1 site (there was no information about the type for 1 site); raw, untreated wastewater samples were collected at these sites using the grab sampling method (a sample collected at a single point in time). A key criterion for selecting these sites was ensuring the likelihood of fecal contamination in the water. During this environmental poliovirus surveillance, collected wastewater samples were transported while maintaining a cold chain of 4–8 °C, using insulated boxes with frozen packs from the collection sites to national poliovirus environmental surveillance (ES) reference laboratory; in cases where shipment was delayed, samples were stored in transit refrigerators at health zone facilities, and frozen packs were replaced prior to shipment to national poliovirus environmental surveillance (ES) reference laboratory.

The data collected by the national reference laboratory for environmental surveillance (ES) include, for each sample taken: the name of the sampling site, province, district, type of wastewater treatment plant or sanitation system, time and date of sampling, dates of shipment to and receipt by the laboratory, sample color and volume, the final cell culture result, and the final ITD and sequencing results.

The implementation of poliovirus ES in the DRC was conducted under a national surveillance framework with technical and logistical support from the World Health Organization (WHO). The Poliovirus Reference Laboratory at the Virology Department of the National Institute for Biomedical Research (INRB) in Kinshasa is currently the only laboratory in the country accredited by the Global Polio Laboratory Network (GPLN). This laboratory conducts analyses of stool specimens collected through acute flaccid paralysis (AFP) surveillance, as well as wastewater samples collected within the ES program for poliovirus detection.

### 2.2. Poliovirus Detection Procedures in Environmental Surveillance

During surveillance activities, wastewater samples were collected using the grab sampling method twice monthly and analyzed at the national environmental surveillance reference laboratory of the INRB. Polioviruses and other enteroviruses were detected using the algorithm recommended by the Global Polio Eradication Initiative (GPEI) guidelines for poliovirus detection in wastewater samples [13]. The process began with concentration of wastewater samples using the two-phase separation method based on dextran and polyethylene glycol (PEG). Specimen (wastewater) volumes were greater than a liter for almost all samples. Sample concentration was initiated immediately upon arrival at the processing laboratory, and no later than 48 h after receipt of the sample. For this concentration step, 0.5 L of wastewater was used, and the remainder was stored at 4 °C (until successful inoculation of the concentrate). Following the protocol, approximately 8 mL of concentrated sample was produced and aliquoted into two tubes (4 mL per tube: one for inoculation and one stored at −20 °C) [13].

Following concentration, concentrated samples were processed for cell culture analysis. Cell culture consisted of inoculating concentrated samples into L20B cell lines, specific for polioviruses, and RD cell lines, sensitive to enteroviruses. Previously, at least five T-25 (25 cm^2^) flasks of L20B monolayer culture and one flask of RD monolayer culture were inoculated with 0.5 mL per flask. Currently, the total number of flasks has been increased to eight (5 L20B and 3 RD) [3,13].

Only culture-positive samples showing cytopathic effects on L20B cell lines and suspected of containing polioviruses underwent molecular characterization. Molecular characterization included intratypic differentiation, real-time reverse transcription polymerase chain reaction (ITD rRT-PCR), and sequencing of the VP1 genomic region to differentiate Sabin-like strains, vaccine-derived polioviruses (VDPVs), and wild polioviruses (WPVs).

Intratypic differentiation is performed using the rRT-PCR ITD kit protocol recommended in the GPLN implemented by the CDC (regularly updated), using 1 µL of the suspected poliovirus isolate (L20B ECP +). The recent version 5.2 of the kit is supplied in a box containing five vials of primers and probes (Quadruplex [EV+Sabin], PanPV, Duplex WPV1/Qβ, PV type 2, WPV3).

When sequencing was required, the isolated culture was dried on Flinders Technology Associates cards and shipped to the NICD in South Africa for Sanger sequencing [14,15].

### 2.3. Evaluation of Site Productivity

The sensitivity of ES sites was assessed using site productivity, defined according to GPEI guidelines as a viral isolation rate of 50% or higher (polioviruses and non-polio enteroviruses). Conversely, a site was considered non-productive if no enteroviruses were isolated for at least six consecutive months. In such cases, the site was closed and replaced by another surveillance site. Overall productivity was calculated as the proportion of enterovirus-positive samples (polioviruses and non-polio enteroviruses) among all wastewater samples collected and analyzed during the study period. Annual productivity was also evaluated at each surveillance site and across all sites.

### 2.4. Statistical Analysis

Data were collected using Epi Info version 3.5.4, exported to Microsoft Excel 365, and analyzed in RStudio version 4.4.1. Descriptive analyses were performed to determine proportions of wastewater samples by surveillance site, province, and year of collection. Annual productivity rates for each site were calculated and visually represented. Trends in poliovirus type detection were expressed as relative frequencies and displayed using bar charts. The spatial and temporal distribution of poliovirus strains was shown through geographic mapping. Additional results were presented using diagrams, graphs, and figures.

## 3. Results

### 3.1. Total Wastewater Samples Analyzed in the Laboratory

**Figure 1:**
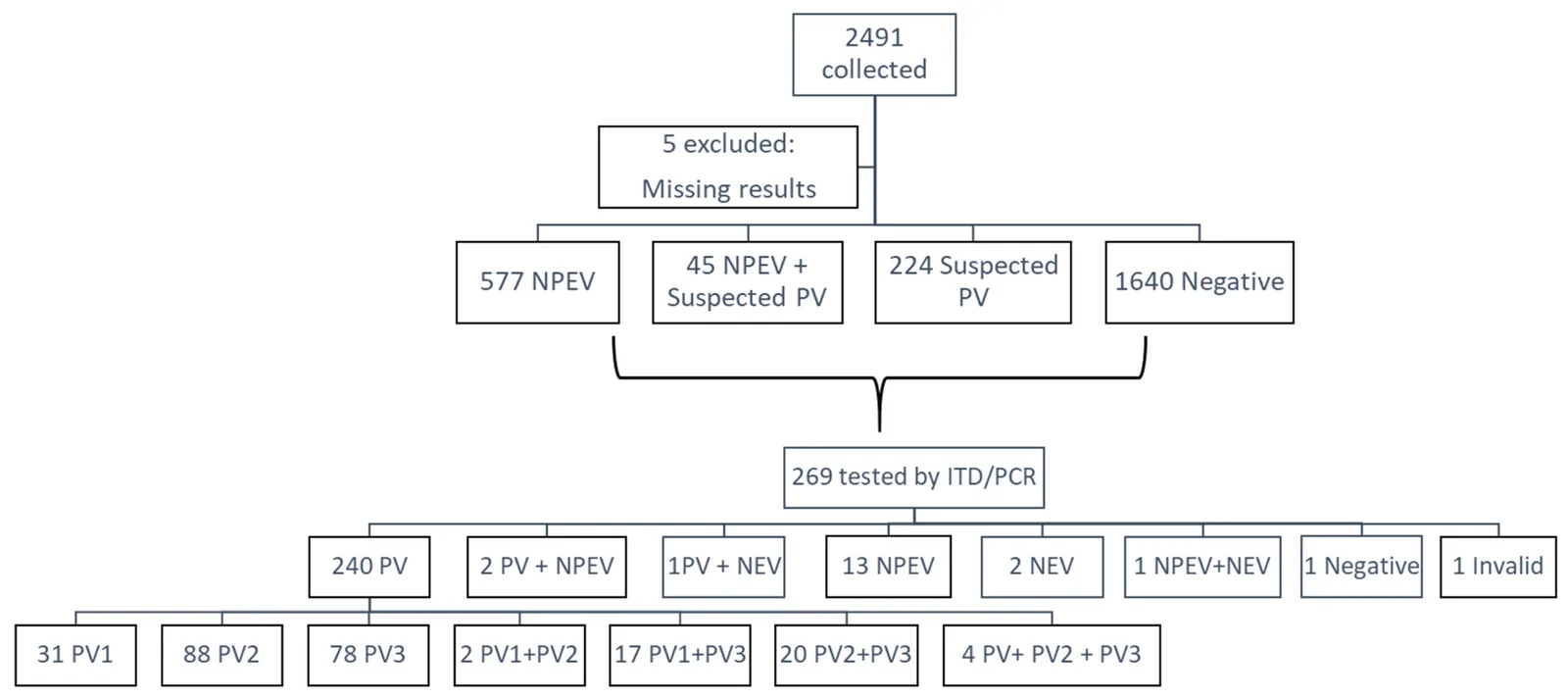
Analysis of wastewater samples for poliovirus environmental surveillance in the Democratic Republic of the Congo, 2017–2025.

A total of 2491 wastewater samples were recorded during the study period. Of these, five samples were excluded due to the lack of laboratory test results. Thus, 2486 samples were selected and included in the statistical analyses (Figure 1). Note that in some samples there was a mixture of enteroviruses or serotypes of poliovirus.

### 3.2. Evolution of the Poliovirus Environmental Surveillance Program in the DRC

Evolution of Environmental Surveillance Sites by Province and Year.

Poliovirus environmental surveillance (ES) in the Democratic Republic of the Congo (DRC) started in 2017 with 6 surveillance sites across three provinces. Over time, the surveillance network progressively expanded, reaching 27 active sites in eight provinces by 2025.

### 3.3. Distribution of Wastewater Samples by Surveillance Site and Province

Surveillance sites were divided into three groups based on sampling activity and collection frequency. Sites with high sampling activity (>130 collected samples) and steady sampling performance included Pakadjuma, André Motors, Baluba, and FIKIN in Kinshasa Province, with 204 (8.2%), 144 (5.8%), 143 (5.7%), and 139 (5.6%) samples collected, respectively. The Camp Assistant site in Haut-Katanga Province recorded 155 samples (6.2%), while the BBL Lulindi and Regideso sites in Maniema Province contributed 149 (6.0%) and 147 (5.9%) samples, respectively.

Other sites showed intermediate activity levels, with sample numbers ranging from 80 to 130 collections. These included the Ecole Française, Pont Moulaert, and Canal Brasserie Ndolo sites in Kinshasa Province, with 138 (5.6%), 123 (4.9%), and 121 (4.9%) samples, respectively, as well as the TP Glodi and Patmos sites in Tshopo Province, with 128 (5.1%) and 127 (5.1%) samples collected, respectively.

Conversely, several sites contributed minimally to the surveillance system. These included Arrêt Béthanie and Rivière Nsanga in Kinshasa Province, with 1 and 36 samples (1.4%), respectively; Bel Air in Haut-Katanga Province with 15 samples (0.6%); Carmel in Lualaba Province with 25 samples (1.0%); and Kapondjo in Maniema Province with 29 samples (1.2%), among others.

### 3.4. Evaluation of Environmental Surveillance Site Sensitivity: Site Productivity

#### 3.4.1. Overall Productivity Based on Sample Analysis

Among the 2486 wastewater samples analyzed by cell culture, enteroviruses—including suspected polioviruses and non-polio enteroviruses—were isolated in 846 samples, resulting in an overall detection rate of 33.9%. Non-polio enteroviruses were the most commonly found viruses, identified in 622 samples (25%). These were isolated alone in 577 samples or alongside suspected polioviruses in 45 samples. Suspected polioviruses were detected in 269 samples (9%), while 1640 samples (66%) showed no viral isolation after cell culture analysis.

#### 3.4.2. Annual Overall Productivity of Environmental Surveillance Sites

**Figure 2:**
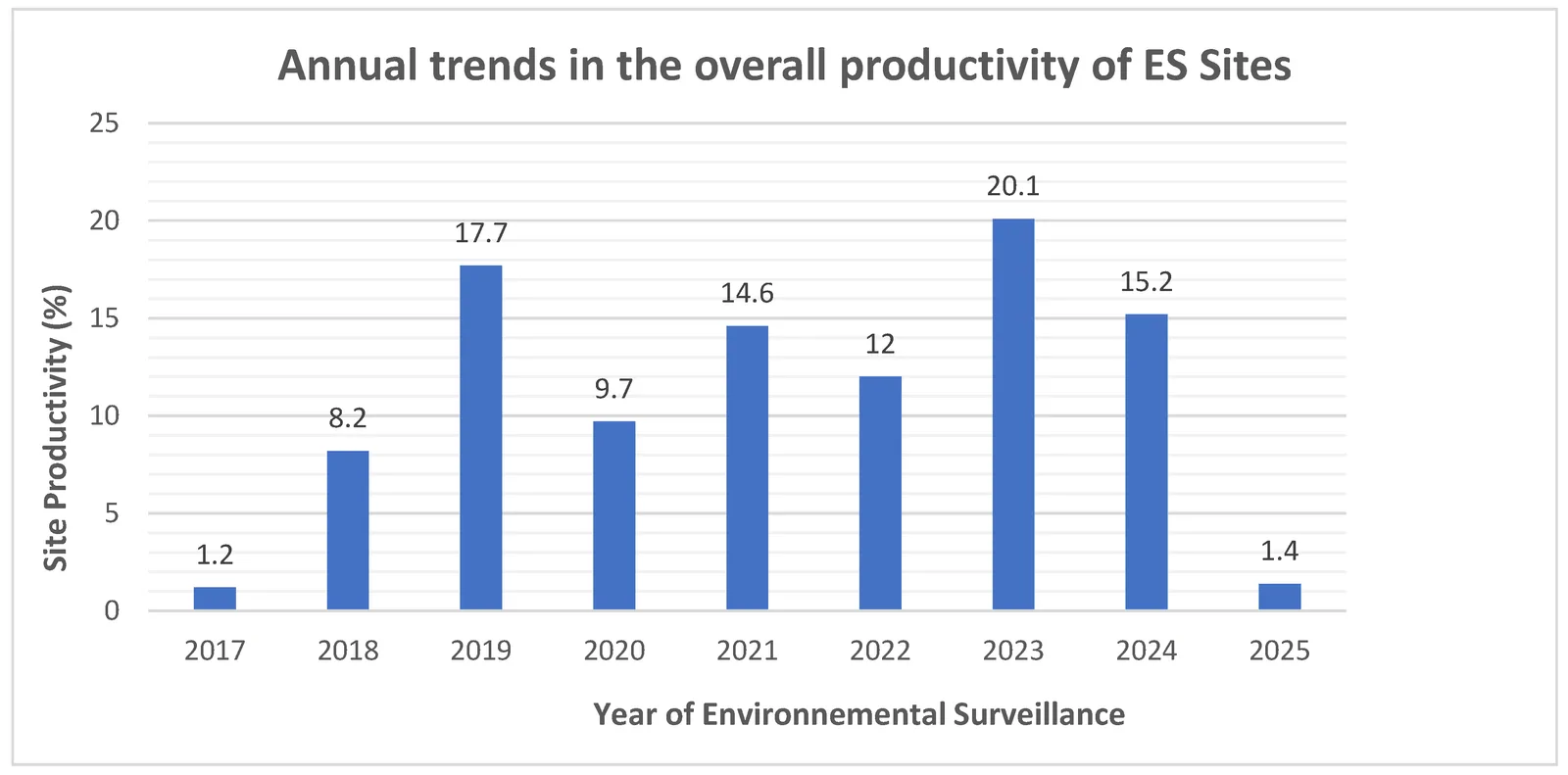
Annual trends in the overall productivity of environmental surveillance sites in the Democratic Republic of the Congo from 2017 to 2025. Productivity is expressed as a percentage.

The annual overall productivity of environmental surveillance sites in the Democratic Republic of the Congo fluctuated between 2017 and 2025, never reaching the recommended 50% threshold. Peaks in productivity peaks were observed in 2019 and 2023, with rates of 17.7% and 20.1%, respectively. Conversely, more significant declines were observed in 2020 and from 2024 onward, with productivity rates of 9.7% and 15.2%, respectively (Figure 2).

#### 3.4.3. Trends in Annual Site Productivity

Most provinces operated with two active environmental surveillance sites, except for Kinshasa, Haut-Katanga, and Maniema, where the maximum number of active sites reached 9, 4, and 3, respectively (Table 1).

Poliovirus environmental surveillance in the Democratic Republic of the Congo showed significant variability in productivity across sites. While some sites frequently exceeded the recommended 50% productivity rate and sometimes approached 100%, there were also periods of very low viral isolation rates (<10%) or no viral detection. These low-productivity periods led to the closure of some sites when such conditions lasted for more than six consecutive months.

Several surveillance sites were discontinued or replaced over time. In Haut-Katanga Province, the Bel Air site was closed and replaced by the Baluba site. In Lualaba Province, the Carmel site was replaced by the Fatshi Béton site. The Pont Kiamvu site in Kongo Central Province was closed without replacement. In Kinshasa Province, the Rivière Nsanga site was replaced by the Arrêt Béthanie site. In Equateur Province, both the Bralima and Pont Royal sites were closed without replacement. Similarly, the Regideso site in Maniema Province and the Ntoluzingu site in Kwilu Province were closed without replacement.

### 3.5. Trends in Poliovirus and Vaccine-Derived Poliovirus Detection in Environmental Surveillance Sites

#### 3.5.1. Identification and Distribution of Isolated Poliovirus Types

**Figure 3:**
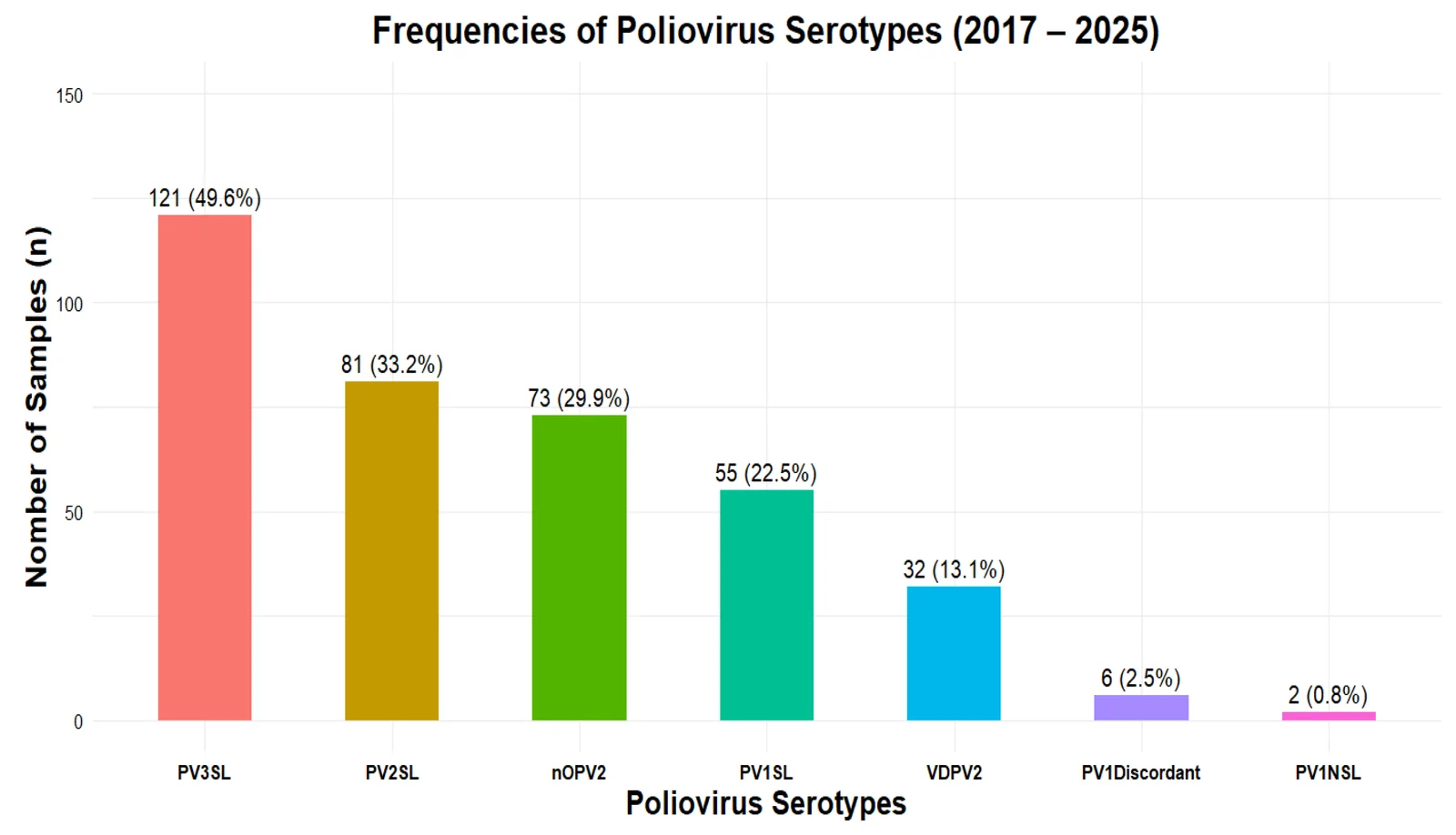
Frequencies of the different poliovirus serotypes detected through environmental surveillance in the Democratic Republic of the Congo. Note: Some wastewater samples contained mixtures of enteroviruses and/or multiple poliovirus serotypes.

Simultaneous circulation of all three poliovirus serotypes (types 1, 2, and 3) was observed, highlighting the coexistence of vaccine-related strains (Sabin-like types 1, 2, and 3; nOPV2) and vaccine-derived strains, particularly type 2 VDPVs. No wild poliovirus (WPV) was detected during the study period (Figure 3).

Among the detected poliovirus strains, serotype 2 viruses, including PV2 Sabin-like strains (PV2SL), VDPV2, and nOPV2, were the most frequently identified, followed by serotype 3 Sabin-like strains (PV3SL), while serotype 1 strains were the least frequently detected.

Among the 269 samples initially identified as suspected poliovirus-positive following cell culture analysis, intratypic differentiation confirmed the presence of polioviruses in 243 samples. Of these confirmed isolates, PV3SL represented the most frequent strain, identified in 121 samples (49.6%), whereas PV1SL was detected in only two samples (0.8%).

#### 3.5.2. Spatial and Temporal Distribution of Polioviruses

Poliovirus circulation exhibited significant spatial and temporal heterogeneity. Kinshasa and Haut-Katanga accounted for 85.2% of viral circulation between 2017 and 2025, with 55.7% and 29.5% of isolates, respectively. In contrast, the remaining five affected provinces, Maniema, Tshopo, Lualaba, Équateur, and Kongo Central, represented only 14.8% of isolates.

The period from 2022 to 2024 was particularly critical, accounting for 88.9% of isolates detected during these three years. A pronounced peak occurred in 2023, representing 53.0% of all isolates identified between 2017 and 2025. This phase was characterized by diversification of circulating serotypes, including the emergence of PV2 nOPV2-like strains from 2022 onward, and a concentration of VDPV2 cases. Notably, 78.1% of all VDPV2 cases recorded in 2022–2023 occurred mainly in Kinshasa. Geographic spread also intensified, increasing from two active provinces in 2017 to a maximum of seven provinces and 20 health zones affected in 2023 (See Supplementary Figure S6 for additional details).

## 4. Discussion

Environmental surveillance of poliovirus through wastewater monitoring has become an important complementary tool for identifying circulating poliovirus strains in the Democratic Republic of the Congo (DRC) and for supporting poliomyelitis control activities alongside acute flaccid paralysis (AFP) surveillance. In the present study, we conducted a comprehensive assessment of ES sites in order to evaluate the evolution, productivity, and sensitivity of the surveillance system since its implementation in the DRC.

### 4.1. Temporal and Spatial Expansion of Environmental Surveillance Sites

The progressive expansion of the poliovirus ES network in the DRC since 2017 reflects a continuous strengthening of national capacities for early detection of poliovirus circulation. The increase from 6 to 27 surveillance sites between 2017 and 2025 demonstrates the country’s growing commitment to implementing the strategies recommended by the Global Polio Eradication Initiative (GPEI). Nevertheless, this expansion has been inconsistent across different provinces and has encountered logistical, financial, and security challenges. The stabilization of surveillance activities within eight provinces from 2021 onward may partly be explained by logistical, financial, and security constraints, but may also reflect a strategic decision to consolidate surveillance performance in high-priority areas. Simultaneously, the continued increase in the number of sites within these provinces suggests a network densification strategy aimed at improving spatial representativeness and overall surveillance sensitivity.

The expansion of the ES network contributed to broader territorial coverage and increased the probability of detecting poliovirus circulation in the environment, which is particularly important for monitoring vaccine-derived polioviruses (VDPVs) and guiding outbreak response activities. This progressive scale-up is consistent with the national poliovirus eradication strategy and aligns with WHO performance recommendations, as previously reported in several countries implementing GPEI-supported environmental surveillance program [6,16,17].

### 4.2. Productivity of Environmental Surveillance Sites

Although the overall productivity rate observed in this study (33.9%) remained below the minimum 50% threshold recommended by GPEI guidelines, the results still show that enteroviruses continue to circulation in the environment.

Overall, the annual productivity of ES sites in the DRC showed marked fluctuations between 2017 and 2025. Following an initial low productivity phase at the end of 2017 (1.2%), productivity progressively increased until 2019 (17.7%), although it remained below the recommended threshold. A decline was subsequently observed in 2020 (9.7%), likely reflecting disruptions related to the coronavirus disease 2019 (COVID-19) pandemic, which may have affected surveillance activities, sample transportation, and laboratory operations. Productivity later improved in 2021 (14.6%) and 2023 (20.1%), before declining sharply again in late 2024 and during the first half of 2025 (1.4%).

Substantial variability in productivity was also observed between surveillance sites. Some sites exceeded the 50% threshold and occasionally reached productivity rates close to 100%, whereas others experienced prolonged periods of very low isolation rates (<10%) or complete absence of viral detection, ultimately leading to site closure after persistent underperformance.

These findings suggest that the ES system in the DRC remains vulnerable to operational, logistical, and contextual challenges. Low viral isolation rates may be associated with several factors, including inappropriate site selection, insufficient fecal contamination of sampling points, suboptimal timing of sample collection, prolonged transport delays, and disruptions in cold-chain maintenance. The quality and stability of environmental surveillance therefore remain highly dependent on operational conditions in both field and laboratory settings.

Previous studies have similarly highlighted the importance of continuous training, regular evaluation, and optimization of site selection for improving ES performance. For example, implementation of environmental surveillance in metropolitan districts of South Africa between 2020 and 2023 achieved productivity rates ranging from 50% to 79.7% following strengthened operational monitoring and training activities [17].

### 4.3. Detection and Circulation of Polioviruses

Despite the relatively low overall productivity observed in this study, vaccine-derived polioviruses, predominantly type 2 VDPVs, accounted for 13.1% of poliovirus isolates following culture confirmation. These findings indicate sustained poliovirus circulation in the environment and further emphasize the importance of environmental surveillance for poliovirus detection.

In many countries where ES has been implemented, wastewater surveillance has provided valuable epidemiological information and early warning signals regarding VDPV circulation. In Egypt, for example, environmental surveillance conducted between 2004 and 2010 identified several VDPV strains in wastewater samples, with almost all isolates belonging to serotype 2, which remains the serotype most frequently associated with circulating VDPV outbreaks globally [18].

Similarly, an environmental viral surveillance study conducted in Japan between April 2010 and January 2013 demonstrated the presence of polioviruses in communities, with serotype 2 being the most frequently isolated (46%) [19].

The WHO has also reported several instances between 2019 and 2020 in which VDPVs were detected through environmental surveillance before, or even in the absence of, confirmed AFP cases in countries such as Afghanistan, Cameroon, Côte d’Ivoire, Egypt, Ghana, Kenya, Liberia, Iran, Senegal, and Chad [10]. In 2024, circulating vaccine-derived poliovirus type 2 (cVDPV2) was detected in wastewater samples in five European countries (Finland, Germany, Poland, Spain, and the United Kingdom) in the absence of prior notification of clinical cases [20].

These observations further support the relevance of ES as an early warning system for identifying silent poliovirus circulation before clinically detectable cases occur.

The spatial and temporal distribution of polioviruses observed in the present study revealed marked heterogeneity in virus circulation patterns across surveillance sites. Some sites, including Regideso, BBL Lulindi, Camp Assistant, and Ecole Française, demonstrated particularly intense viral activity between 2022 and 2024. The repeated detection of PV2 Sabin-like strains, PV3 Sabin-like strains, and nOPV2 in multiple sites could suggest continuous virus circulation driven by both vaccination activities and persistent local transmission.

Moreover, the recurrent detection of VDPV2 in several sites over relatively short periods highlights persistent pockets of vulnerability within certain communities could plausibly indicate insufficient vaccine coverage. This period of intensive detection (2022–2024) coincided with the time when national polio vaccination coverage in the DRC, according to WHO and UNICEF estimates of national immunization coverage (WUENIC) was at its lowest: 61% in 2023, 65% in 2022 and 2024, and 66% in 2021 [21]. It should be noted that, throughout the period studied, national vaccination coverage ranged from 72% to 61%, a rate well below the 95% threshold set to prevent the emergence of circulating vaccine-derived polioviruses (cVDPVs) [22].

Our findings therefore not only demonstrate the persistence of poliovirus circulation in densely populated urban settings, but also illustrate the capacity of ES to identify potential transmission hotspots and support outbreak monitoring activities.

Importantly, no wild poliovirus was detected throughout the study period. These findings reinforce the hypothesis that the DRC remains free from documented wild poliovirus circulation and indirectly support the effectiveness of current poliovirus containment and eradication strategies in the country. Similar observations were reported by Blomqvist et al. in Egypt, where systematic environmental surveillance conducted since 2000 identified no cases of poliomyelitis caused by wild poliovirus after 2005.

### 4.4. Study Limitations

This study has several limitations that should be considered when interpreting the findings. First, the analysis was based on routinely collected surveillance data, which may have been affected by incomplete records and variations in data standardization and operational practices over time. Second, environmental surveillance activities were conducted in only 8 of the 26 provinces of the Democratic Republic of the Congo during the study period, which may limit the national representativeness of the findings and the ability to detect poliovirus circulation in non-surveyed areas.

In addition, the marked variability in site productivity observed across provinces and over time likely reflects operational and logistical constraints, including site selection, sampling quality, transport conditions, and maintenance of the cold chain. Furthermore, the INRB laboratory in Kinshasa remains the only WHO-accredited poliovirus laboratory in the country, which may contribute to delays in sample processing, increased analytical workload, and system vulnerability in the event of major disruptions. Finally, surveillance sensitivity was assessed primarily through site productivity (viral isolation rate ≥50%), an indicator recommended by GPEI guidelines but which may not fully reflect the actual capacity of the system to detect low-level or early poliovirus circulation.

Despite these limitations, this study remains highly important because it provides one of the first comprehensive evaluations of poliovirus environmental surveillance implementation in the DRC over an extended period. In a country characterized by recurrent cVDPV outbreaks, heterogeneous vaccination coverage, and major logistical challenges, these findings provide valuable operational and epidemiological evidence to guide surveillance strengthening, optimize site selection, and support poliovirus eradication efforts.

### 4.5. Study Strengths

This study presents several important strengths. It documents eight years of poliovirus environmental surveillance implementation in the DRC, providing valuable longitudinal data on the evolution and performance of the surveillance network. The study also highlights the important contribution of environmental surveillance in detecting vaccine-derived polioviruses while supporting the absence of documented wild poliovirus circulation during the study period.

In addition, the analysis of site productivity and viral detection patterns provides practical insights to improve surveillance quality, guide geographic expansion, and strengthen operational performance. Conducted in one of the largest and most logistically challenging countries in Africa, this work further demonstrates the relevance and feasibility of environmental surveillance as a complementary tool to AFP surveillance in high-risk settings. Finally, the findings may provide useful insights for other low- and middle-income countries facing similar challenges during the final stages of global poliovirus eradication efforts.

## 5. Conclusions

This study emphasizes the significant role of environmental surveillance (ES) in poliovirus monitoring in the DRC. From 2017 to 2025, the number of surveillance sites increased from 6 to 27, and 2486 wastewater samples were analyzed. Enteroviruses were found in 33.9% of samples, with annual detection rates remaining below the 50% target (peaking at 20.1% in 2023). Vaccine-derived poliovirus type 2 (VDPV2) accounted for 13.1% of poliovirus isolates, with no wild poliovirus found. Our findings confirm the importance of ES in identifying circulating vaccine-derived strains and supporting the absence of wild poliovirus. However, ongoing fluctuations in site productivity highlight the need to enhance operational performance. Maintaining high vaccination coverage, improving site productivity, and responding quickly to detections are critical to advancing polio eradication efforts in the DRC.

## Figures and Tables

**Table 1:** Annual site productivity.

Province	Site	2017	2018	2019	2020	2021	2022	2023	2024	2025
Haut-Katanga	Camp Assistant ^∞^	75	44	73.1	60	55.6	50	68	41.7	16.7
	Bel Air ^↓^	25	0	X	X	X	X	X	X	X
	Baluba ^∞↗^	—	61.5	80.8	72.7	72.2	57.1	66.7	37.5	16.7
	Canal Brondo	—	—	—	—	—	—	28.6	34.8	0
	Kanavyondo	—	—	—	—	—	—	28.6	34.8	0
Kinshasa	André Motors ^∞^	25	29.2	38.5	38.5	25	44.4	50	45.8	0
	Paka Djuma ^∞^	50	58.3	65.4	60	58	25	79.2	36	42.9
	Ecole Française ^∞^	—	50	26.9	23.1	33.3	21.7	31.8	25	0
	FIKIN ^∞^	—	70	16	23.1	0	26.1	54.2	41.7	25
	Pont Moulaert ^∞^	—	—	14.3	30	8.3	54.2	54.2	29.2	25
	Canal Brasserie Ndolo ^∞^	—	—	0	9.1	25	52.2	56.5	33.3	12.5
	Canal Pont Matete ^∞^	—	—	—	—	—	—	60	29.2	12.5
	Canal Quartier Kutu ^∞^	—	—	—	—	—	—	60	29.2	0
	Rivière Nsanga ^↓^	—	—	—	—	—	—	40	25	0
	Arrêt Béthanie ^↗^	—	—	—	—	—	—	—	—	0
Maniema	BBL Lulindi ^∞^	50	26.1	60	54.5	52.2	19	25	5.6	0
	Regideso ^∞↓^	25	39.1	68	72.7	47.8	40	42.1	10	0
	Kapondjo	—	—	—	—	—	—	20	10	0
Tshopo	Patmos ^∞^	—	100	68	53.8	36.4	22.7	26.1	16.7	0
	TP Glodi ^∞↓^	—	100	64	76.9	38.5	9.1	21.7	13	0
Kongo Central	Bilawumba ^∞^	—	—	50	41.7	33.3	13.3	18.8	10.5	0
	Pont Kiamvu ^∞↓^	—	—	100	41.7	33.3	6.7	23.5	5.3	0
Lualaba	Bassin SNCC ^∞^	—	—	—	0	37.5	50	15.4	9.5	0
	Carmel ^↓^	—	—	—	0	33.3	0	X	X	X
	Pont FATSHI Béton ^↗^	—	—	—	—	—	0	28.6	14.3	0
Equateur	BRALIMA ^↓^	—	—	—	0	18.2	16.7	27.3	0	0
	Pont Royal ^∞↓^	—	—	—	0	16.7	8.3	20	0	50
Kwilu	Ntoluzingu ^↓^	—	—	—	—	28.6	10	7.1	0	0
	Lui	—	—	—	—	28.6	30	8.3	6.3	0

Legend: ^∞^: Site having achieved a productivity rate of 50% at least once, during the period of our study. ^↓^: Site closed due to lack of productivity. ^↗^: Replacement site (of a closed site). X: No more data to collect (site closed).

## Data Availability

All collection and laboratory analysis data are available upon request from the corresponding and principal authors.

## Supplementary Materials

The following supporting information can be downloaded at: https://www.mdpi.com/article/10.3390/pathogens15090917/s1, Figure S1: Evolution of province and site numbers opened in RDC from 2017 to 2025; Figure S2: Distribution of samples by ES sites and province in DRC; Figure S3: Overall productivity in terms of samples: (a) Cell culture results et (b) Overall productivity rate; Figure S4: Trends (2017–2025) in productivity per PV ES sites in DRC: Each plot relates to a set of sites by provinces: [1] Sites from Kinshasa; [2] Sites from Maniema and Tshopo; [3] Sites from Equateur, Kwilu and Kongo Central et [4] Sites from Haut-Katanga and Lualaba; Figure S5: Temporal and geographic distribution of PV serotypes per province, as detected in DRC from ES; Figure S6: Annual trend in circulation of PV serotypes in DRC as detected by ES; Figure S7: Annual trends of PV serotype detected in DRC from ES.

## Abbreviations

AFP	Acute flaccid paralysis
cVDPV	circulating Vaccine-derived polioviruses
DRC	Democratic Republic of the Congo
ES	Environmental Surveillance
GPEI	Global Polio Eradication Initiative
GPLN	Global Polio Laboratory Network
INRB	Institute National de Recherche biomédicale
IPC	Expanded Programme on Immunization
NEV	Non enterovirus
nOPV2	Novel oral polio vaccine type 2
NPEV	Non-Poliovirus enterovirus
PV1	Poliovirus serotype 1
PV1SL	Poliovirus serotype 1 Sabin Like
PV2	Poliovirus serotype 2
PV2SL	Poliovirus serotype 2 Sabin Like
PV3	Poliovirus serotype 3
PV3SL	Poliovirus serotype 3 Sabin Like
WHO	World Health Organization
WPV1	Wild poliovirus serotype 1
