# Supplementary material for: Environmental Surveillance of Poliovirus in the Democratic Republic of the Congo: Sensitivity and Site Productivity, 2017–2025
Source: Pathogens. 2026 Aug 31;15(9):917. doi: 10.3390/pathogens15090917 (PMC13610432; doi:10.3390/pathogens15090917)
Supplement: Supplementary file 1 [file pathogens-15-00917-s001.zip › pathogens-4375169-supplementary-update.pdf]

SUPPLEMENT MATERIALS

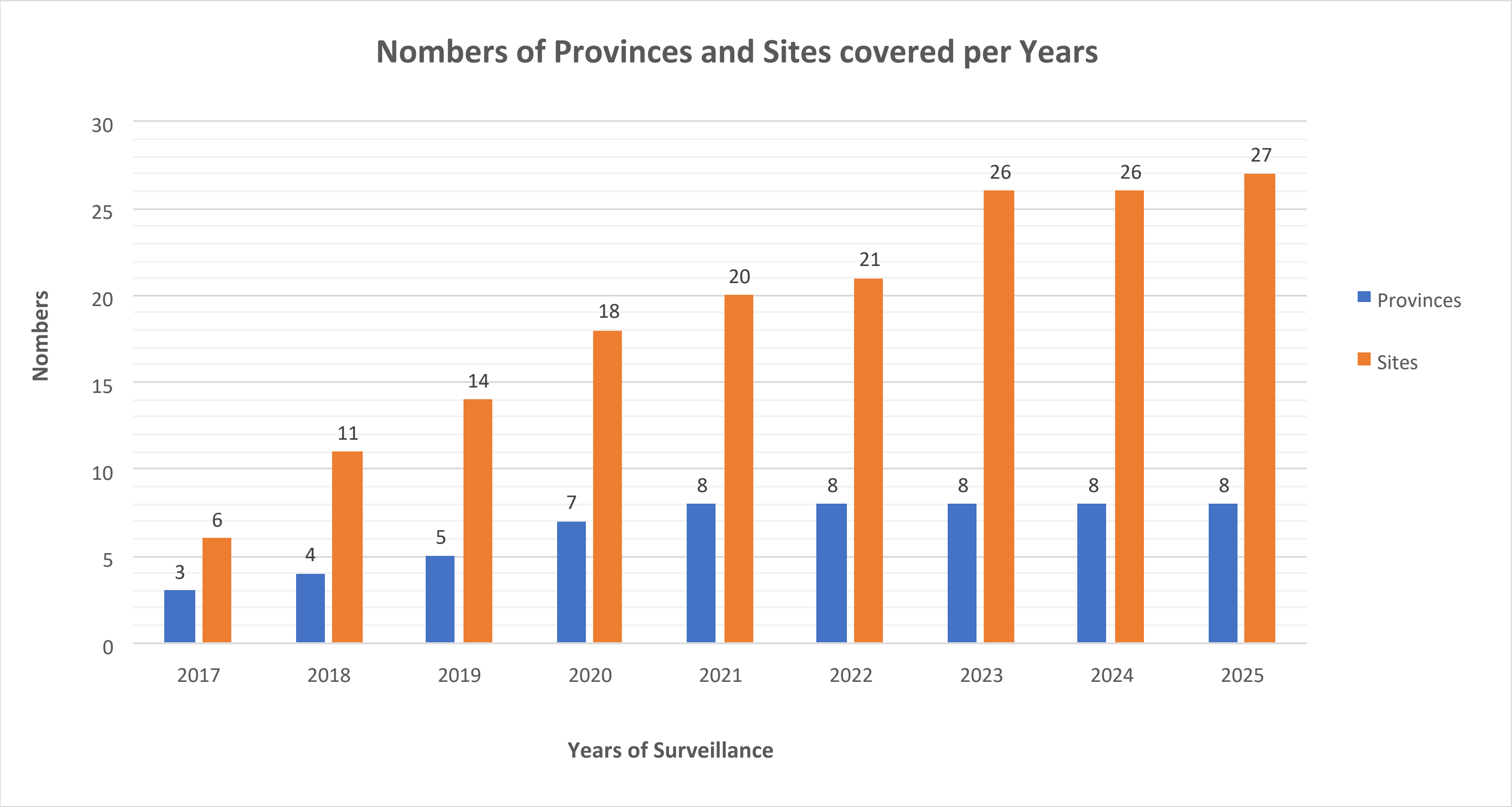

figure S 1Evolution of province and site numbers opened in RDC from 2017 to 2025

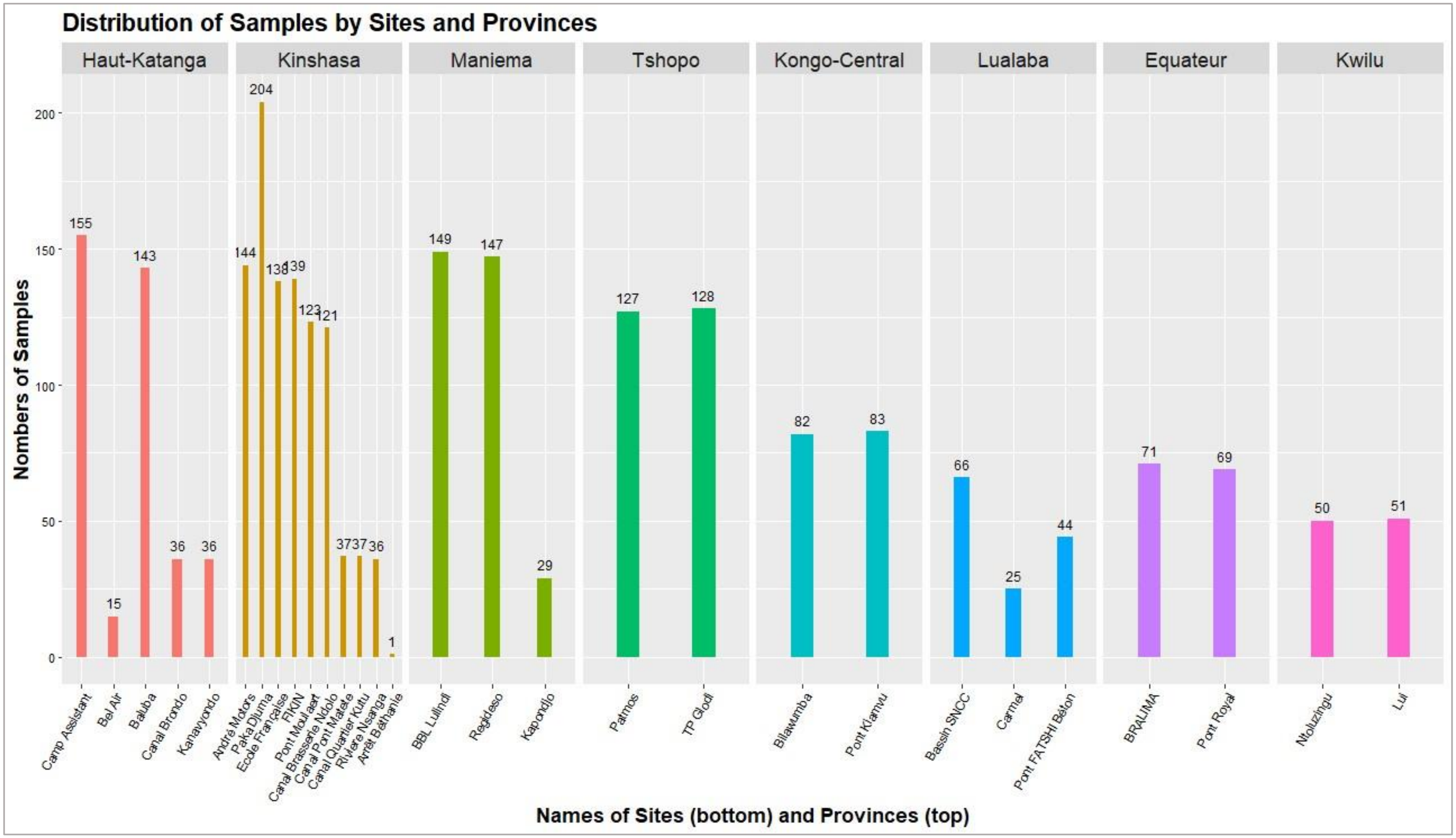

figure S 2Distribution of samples by ES site and province in DRC

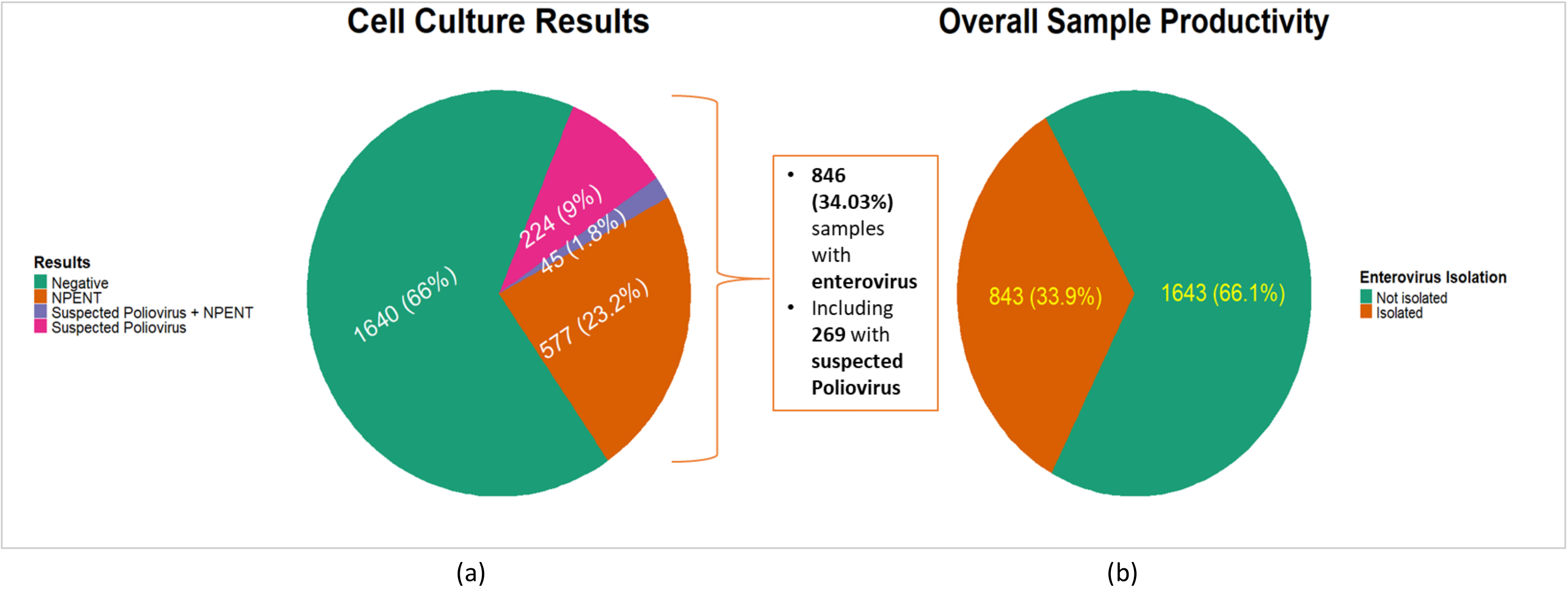

figure S 3Overall productivity in terms of samples: (a) Cell culture results et (b) Overall productivity rate

(1) (2)

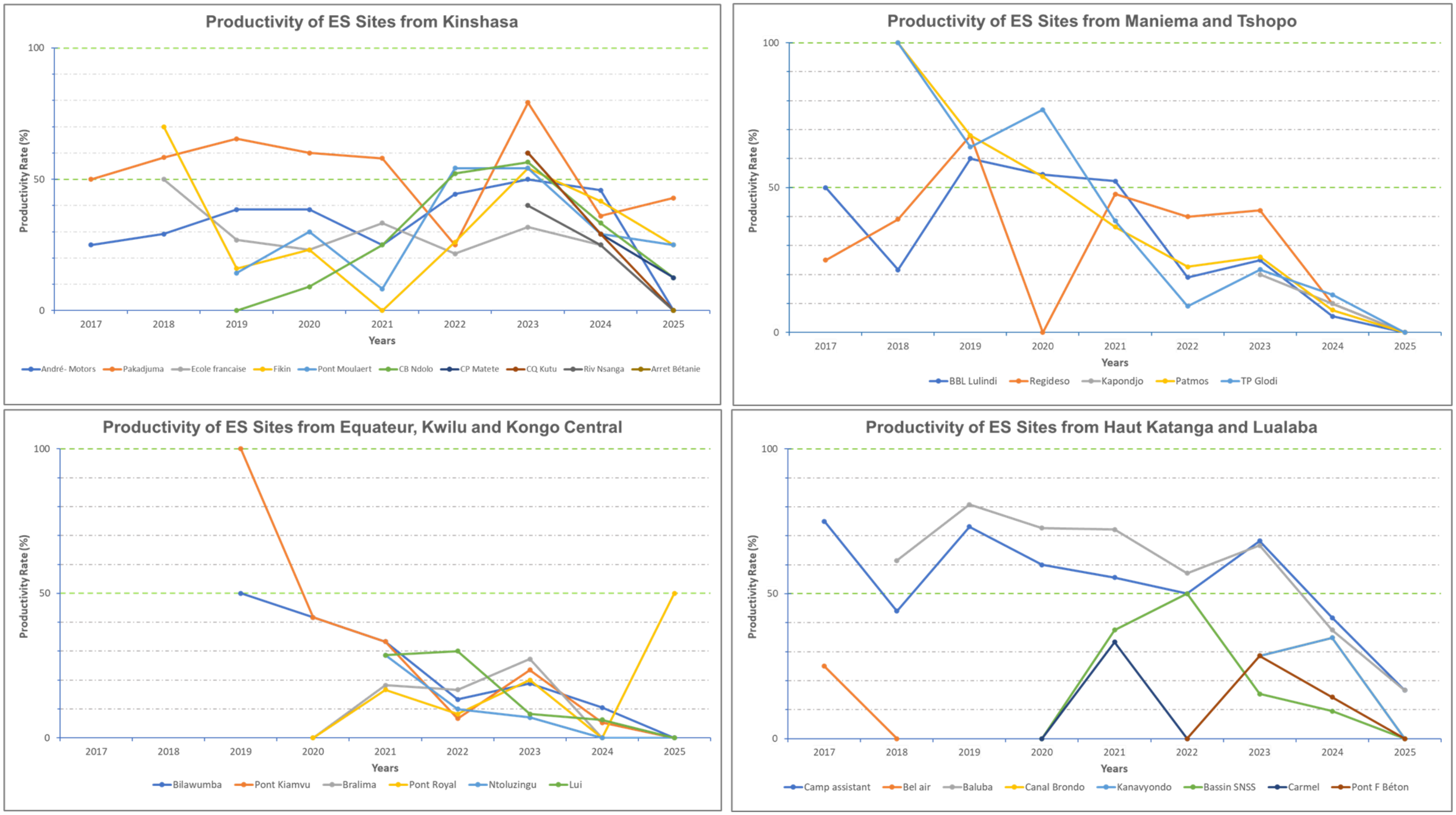

(3) (4)

figure S 4 Trends (2017–2025) in productivity per PV ES sites in DRC: Each plot relates to a set of sites by provinces: [1] Sites from Kinshasa; [2] Sites from Maniema and Tshopo; [3] Sites from Equateur, Kwilu and Kongo Central et [4] Sites from Haut-Katanga and lualaba

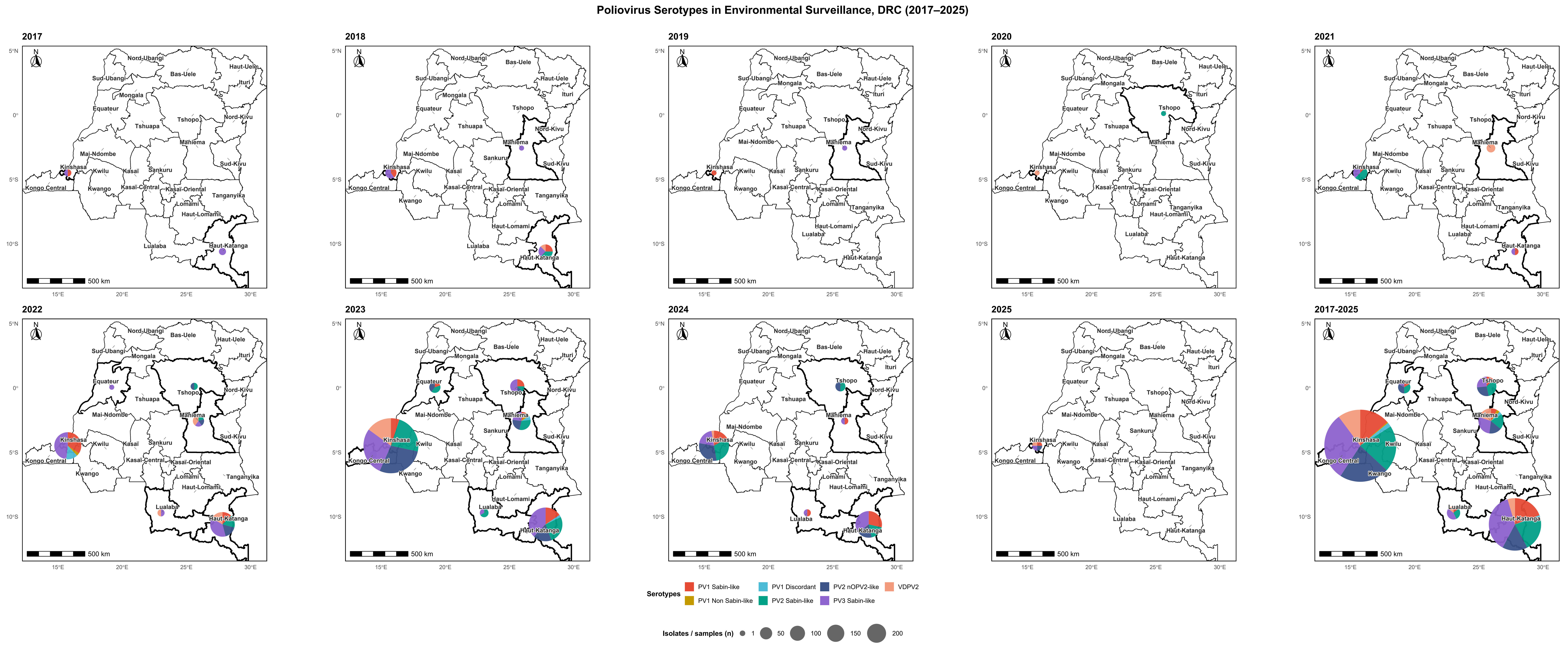

Figure S5. Temporal and geographic distribution of poliovirus serotypes detected through environmental surveillance in the Democratic Republic of the Congo, 2017–2025. Each map shows the serotype composition by province for the indicated year; the final map summarizes the entire study period. Pie size reflects the number of samples. Maps were generated using RStudio

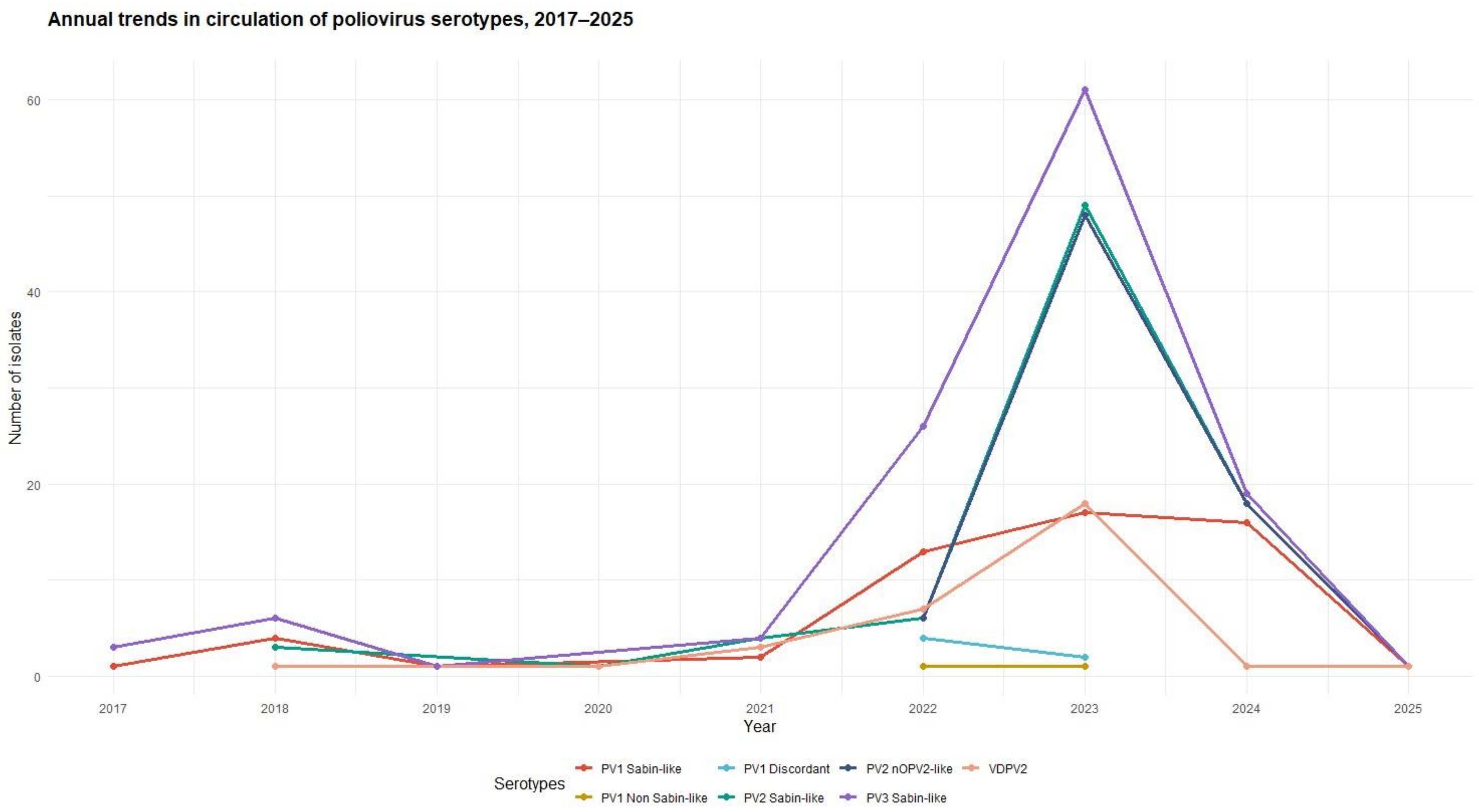

figure S 6: Annual trend in circulation of PV serotypes in DRC as detected by ES

.

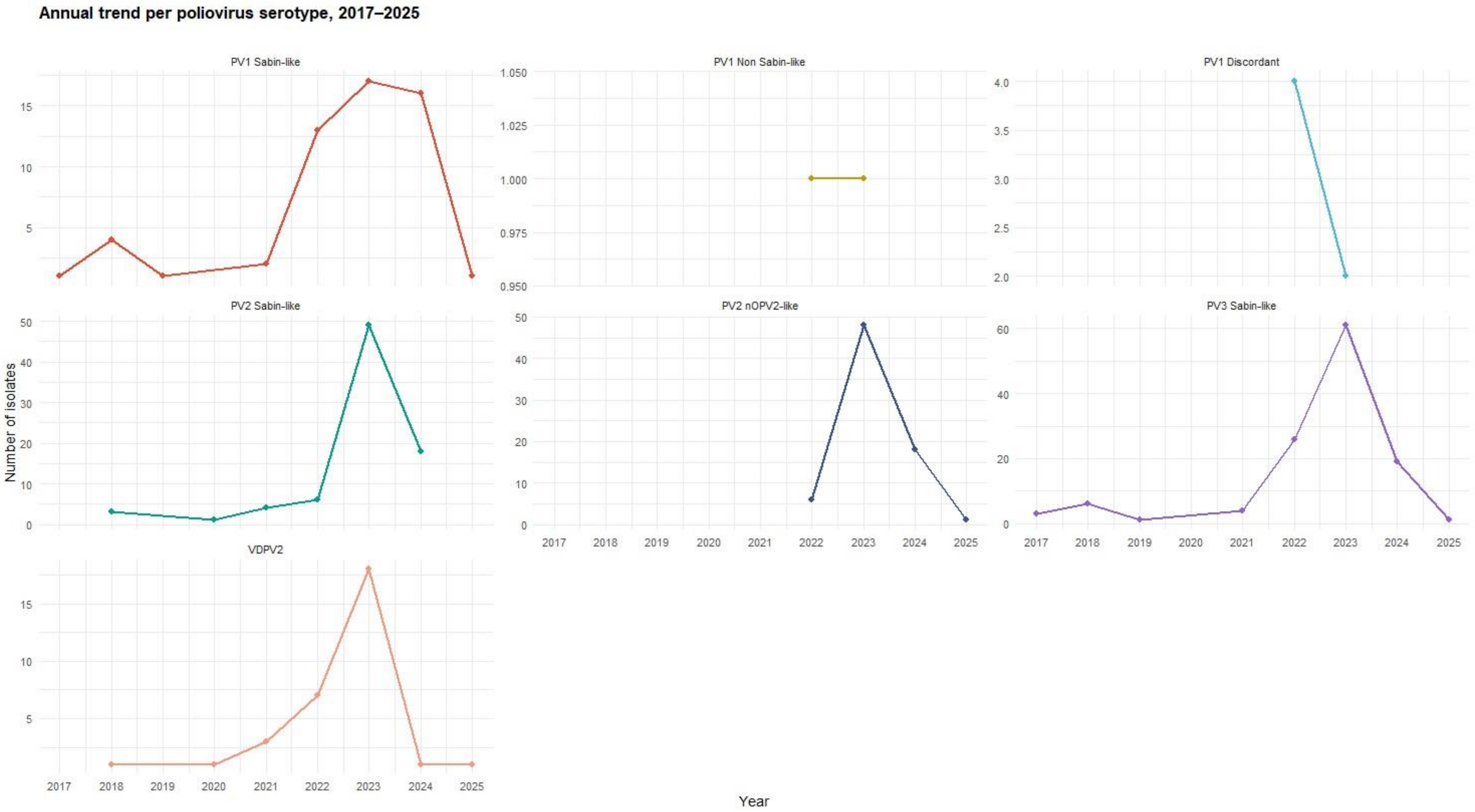

figure S 7Annual trends of PV serotype detected in DRC from ES
